# Binding pocket dynamics along the recovery stroke of human β-cardiac myosin

**DOI:** 10.1371/journal.pcbi.1011099

**Published:** 2023-05-18

**Authors:** Fariha Akter, Julien Ochala, Arianna Fornili

**Affiliations:** 1 Department of Chemistry, School of Physical and Chemical Sciences, Queen Mary University of London, London, United Kingdom; 2 Department of Biomedical Sciences, University of Copenhagen, København N, Denmark; 3 Centre of Human and Applied Physiological Sciences, King’s College London, London, United Kingdom; Danish Cancer Society Research Center, DENMARK

## Abstract

The druggability of small-molecule binding sites can be significantly affected by protein motions and conformational changes. Ligand binding, protein dynamics and protein function have been shown to be closely interconnected in myosins. The breakthrough discovery of omecamtiv mecarbil (OM) has led to an increased interest in small molecules that can target myosin and modulate its function for therapeutic purposes (myosin modulators). In this work, we use a combination of computational methods, including steered molecular dynamics, umbrella sampling and binding pocket tracking tools, to follow the evolution of the OM binding site during the recovery stroke transition of human β-cardiac myosin. We found that steering two internal coordinates of the motor domain can recapture the main features of the transition and in particular the rearrangements of the binding site, which shows significant changes in size, shape and composition. Possible intermediate conformations were also identified, in remarkable agreement with experimental findings. The differences in the binding site properties observed along the transition can be exploited for the future development of conformation-selective myosin modulators.

## Author summary

Small molecules, including drugs, tend to bind to crevices or pockets on the surface of proteins. The shape, size and composition of these binding sites can change as a result of the protein dynamics. This is particularly important if the motions that modulate the binding site are linked to the function of the protein, since molecules that can selectively target different binding site states will likely have different effects on the protein function. In this work we focus on β-cardiac myosin, a key protein for heart contraction that has become the subject of intensive research in recent years because of the therapeutic potential of its small-molecule modulators. Using computational techniques, we show how the binding site of the first-in-class cardiac myosin activator omecamtiv mecarbil changes during the recovery stroke, one of the main functional motions of the protein. Importantly, we identify features in the shape, size and composition of the site that can be exploited for the development of drugs that can target different stages of the recovery stroke and thus have different types of biological effects.

## Introduction

The ability of proteins to bind small molecules can be significantly affected by protein dynamics. Binding sites can be modified by sub-pockets or adjacent pockets that open and close as a result of thermal fluctuations [1]. In some cases, binding pockets are fully formed only in some of the conformations adopted by the protein [2,3]. Knowledge of protein dynamics can thus be exploited in drug development pipelines to detect and target binding pockets that would be overlooked if only single protein structures were considered [4–6].

**Fig 1:**
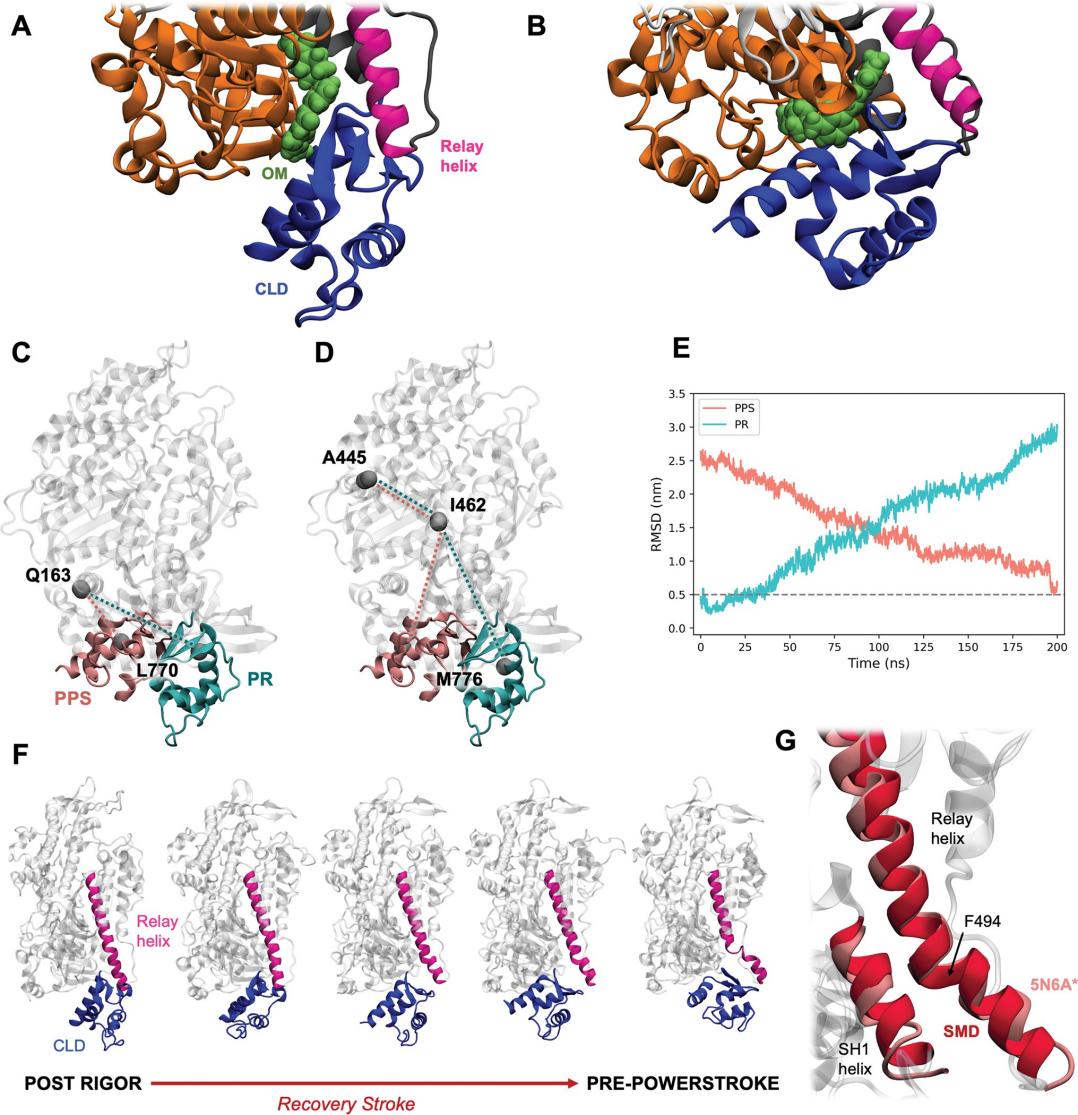
Modelling the recovery stroke. **A/B.** Close-up view of the OM binding site in PR (A) and PPS (B) structures of cardiac myosin (cartoon) in complex with OM (spheres). The following colour code was used for the different sub-domains: N-terminal domain: orange, L50K: grey, U50K: white, relay helix: magenta, converter and lever arm (CLD): blue. **C/D.** The atoms used to define the collective variables D1 (C) and A1 (D) are represented as grey spheres and connected with dotted lines. The CLD is shown as coloured cartoon in the PR (cyan) and PPS (pink) conformation, while the rest of the protein is shown in white. **E.** Time evolution of the CLD RMSD from the reference PR (4P7H*, cyan) and PPS (5N6A*, pink) conformations during the 200-ns apoPR-to-PPS SMD simulation. The RMSD was calculated over the C_α_ atoms of the CLD domain after a best-fit superimposition of the C_α_ atoms of the rest of the protein. **F.** Snapshots extracted from the 200-ns apoPR-to-PPS SMD simulation in panel E, showing the rotation of the CLD domain (blue) and the formation of the kink in the relay helix (magenta). **G.** Close-up view of the SH1 and relay helices at the end of the 200-ns PR-to-PPS SMD trajectory (red), superimposed to the reference PPS structure (pink).

This work focuses on myosin II, a key protein for muscle contraction that undergoes extensive conformational changes during its functional cycle [7]. Changes in the nucleotide and actin-binding sites of myosin are coupled to large swinging motions of the converter and lever arm domain (CLD in the following), which can alternatively adopt a ‘down’ (post-rigor or PR, Fig 1A) and ‘up’ (pre-power stroke or PPS, Fig 1B) conformation.

The motor domain of myosins has been found to contain multiple small-molecule binding sites [8]. One of them is located at the interface between the CLD and the rest of the protein (Fig 1A and 1B), so that it undergoes large rearrangements when myosin switches from the down to up state (recovery stroke) or vice versa (power stroke). This site is known to be bound by omecamtiv mecarbil (OM), a first-in-class cardiac myosin modulator that increases force production in cardiac myocytes and has been investigated in clinical trials for the treatment of heart failure [9–11]. OM has been shown to bind myosin in both the PR [12] (Fig 1A) and the PPS [13] (Fig 1B) conformations. While its affinity for the PPS state is stronger [13,14], and its mechanism of action has been linked to its ability to stabilise the PPS and subsequent states in the actomyosin cycle [13,15], the existence of a OM-bound PR structure indicates that the site can be targeted by small molecules also in the PR state.

In this work, we use multiple computational techniques to model the evolution of the OM binding site along the PR-to-PPS transition (recovery stroke) of human β-cardiac myosin. We show that the binding pocket gradually transitions from the PR to the PPS shape, with different regions being recruited to the site or excluded from it as the CLD rotates upward. This change is accompanied by an increase in pocket size and polarity. Evaluation of the free energy along the recovery predicts the existence of stable intermediates that could be selectively targeted to produce myosin modulators with new properties.

## Methods

### System preparation

Molecular Dynamics (MD) simulations were carried out on the human β-cardiac myosin motor domain (residues 1 to 783) using GROMACS (2016.6 version) [16]. The starting structures for the simulations were generated from X-ray structures of cardiac myosin in the PR (PDB ID: 4P7H [12]) and PPS (PDB ID: 5N6A [13]) state as described in Ref. [17] (PR) and Ref. [14] (PPS). The starting structures subjected to energy minimisation will be indicated with the PDB ID used for their generation followed by a * symbol and used as references in some of the analyses.

Simulations were run both in the absence (apoPR) and presence (PR and PPS) of Mg-ADP.Pi. For each system, the protein was described with the AMBER ff99SB*-ILDN force field [18], while the nucleotide was described using parameters from Ref. [19]. The system was solvated using a truncated octahedral box of TIP3P water molecules, using a minimal distance of 1.5 nm between the protein and the walls of the box. The charge of the ionisable residues was set to that of their standard protonation state at pH 7 and the system was neutralised by adding counter-ions. Energy minimization and equilibration to T = 300 K and p = 1 bar were run with the protocol and parameters described in Ref. [14] and [17].

Since the X-ray structure for the PR state 4P7H does not include a nucleotide, the following procedure was used to add it to the starting structure of the PR simulations. A preliminary system was first prepared by transferring Mn-AMPPNP from the PDB structure 4DB1 and converting it into Mg-ATP. Energy minimisation and equilibration were performed using the same protocol as for apoPR, except for the last equilibration step, which was extended to 100 ns. Mg-ATP was then converted into Mg-ADP.Pi and the resulting system was subjected to a second round of energy minimization and equilibration.

### Collective variable identification

Defining correct collective variables is a crucial step to sample reliable conformational transitions with enhanced sampling. These geometrical parameters must have significantly different values between the two end point states, here being the PR and PPS states. Previous MD studies on myosin have mostly used the Root Mean Square Deviation (RMSD) from the endpoints to drive the recovery stroke [20–23]. In order to avoid distortions in the local geometry of the protein, especially in the side chains of the binding site, an alternative set of CVs was adopted in this work. Candidate CVs were first identified using two 300-ns long unbiased MD trajectories of PR [17] and PPS [14] states of human β-cardiac myosin. Different distances, angles and dihedral angles were tested. Angles were selected to capture the rotation of the converter and lever arm domain (CLD, residues 711 to 783) during the recovery stroke. They were paired to distances between the CLD and the rest of the protein to provide the correct directionality to the motion. A total of 16 distances and 11 angles were tested. The D1 (Q163 and L770) and D2 (I585 and K707) C_α_-atom distances were the best in terms of PR vs PPS separation and stability of values during the simulations (Fig A in S1 Text). Similarly, the angle A1 (formed by A445, I462 and M776 C_α_ atoms) had the largest gap in values between PR and PPS (Fig B in S1 Text). A further set of angles was tested to measure the kink of the relay helix during the recovery stroke, and the angle A2 (formed by L485, M493 and E500 C_α_ atoms) was found to be the best. The four CVs—D1, D2, A1 and A2—were paired in 6 different combinations (Fig C in S1 Text). The best combination was then selected as that leading closest to the PPS state in Steered Molecular Dynamics (SMD) simulations (see below).

### SMD simulation protocol

Preliminary SMD simulations were initially performed starting from the apoPR state to test the ability of different combinations of CVs to drive the recovery stroke. The best combination of CVs was then used to simulate both the PR-to-PPS (recovery stroke) and PPS-to-PR (inverse recovery stroke) transition (Table A in S1 Text).

SMD simulations were run using GROMACS and the PLUMED [24] plugin (version 2.4.6). Moving restraints were applied on each CV using harmonic potentials, where the reference value in the biasing potential was varied from an initial to a final value in 20 (short SMD runs) or 200 (long SMD runs) ns. Initial values were taken from the equilibrated structure of the starting state, while the final values were calculated as averages over unbiased MD simulations of the target state [14] (Table A in S1 Text). The value of the force constant was set to 5000 kJ/mol/nm^2^ (distances) or 5000 kJ/mol/rad^2^ (angles).

Short SMD simulations of the recovery stroke were performed on selected CV combinations (Fig D in S1 Text). The combinations leading to the lowest RMSD from the target state (D1, A1 and D2, A1) were then used again in long SMD runs. As expected, both combinations got closer to the target state compared to the short runs, but D1, A1 was found to be the best in terms of RMSD (Fig E in S1 Text) and it was used in all the subsequent calculations.

### MDPocket analysis

The 200-ns SMD trajectory starting from the PR state was analysed with MDPocket [25] to monitor the time evolution of the shape of the pocket and of properties such as volume, polar surface area, polarity and hydrophobicity scores. The trajectory was divided into 20 batches by splitting the CV space into 20 regular intervals (ΔD1 = 0.04 nm, ΔA1 = 0.1 rad). Each batch contained an average of 5000 frames (ranging from about 2400 to 7200) that were aligned using the C_α_ atoms of the protein. For each batch, an isovalue of 0.3 was used for the frequency grid visualization to detect pockets that are present in at least 30% of the frames. If two pockets were observed in the region corresponding to the binding site, both were considered in the calculation of the pocket properties. For each of the representative batches B1, B7, B10, B15 and B20, the frame with the pocket volume closest to the batch average was extracted. For B10, which has two disjoint pockets (*top* and *bottom*), the frame with the lowest RMSD from the average volumes of the two pockets was used. Fpocket was then run on these representative frames to analyse the amino acidic composition of the pocket. In some cases, fpocket identified more than one pocket in the binding site region as defined by the MDpocket frequency grids. All the pockets found in this region were considered in our analysis.

### US simulations and WHAM-2D

US simulations were performed to estimate the free energy change along the transitions sampled by the 200ns PR-to-PPS (recovery stroke) and PPS-to-PR (inverse recovery stroke) simulations using D1 and A1 as CVs. The value of force constant applied to each CV for each window was calculated as

$$k_{US}=\frac{k_{B}T}{\sigma^{2}}$$

where *k_B_* is the Boltzmann constant, *T* the temperature and *σ*^2^ the variance of the CV estimated from unbiased MD simulations [17]. This resulted in a *k_US_* value of 300 kJ/mol/nm^2^ for D1 and of 1000 kJ/mol/rad^2^ for A1.

Preliminary simulations were run to determine the number and location of windows to be used to cover the low energy regions of the landscape. Windows were added and simulations were extended until no significant change in each free energy landscape was observed. A total of 22 (recovery stroke) and 23 (inverse recovery stroke) windows were used (Tables B and C in S1 Text). For each window, the initial part where the CVs were equilibrating was discarded and simulations were extended to have at least 90 ns of production for the Weighted Histogram Analysis Method (WHAM) analysis. A total of 2,685 and 2,690 ns was run for the PPS-to-PR and PR-to-PPS landscapes, respectively. Starting structures were extracted from 200-ns long SMD trajectories run with the best CV combination (D1, A1). For each window, the SMD frame with CV values closest to the window restraints was selected. This method was preferred to the alternative approach of using the last structure of the previous window because in preliminary calculations less time was required to equilibrate the system around the given restraint.

The results from the different windows were combined to reconstruct the free energy landscape by using the 2D-WHAM method (WHAM 2.0.9 version [26]). The convergence tolerance, the number of histogram bins and the temperature were set to 0.000001 kJ/mol, 20 and 300 K respectively. Results of WHAM were visualised and further analysed using Python code to locate low-energy regions.

Representative structures of local minima in the energy landscape were extracted by cluster analysis. All the frames from US simulations mapped to each local minimum (CV values within the 2D bin from WHAM corresponding to the local minimum) were first collected. A cluster analysis was then performed on each set of frames using the *gromos* [27] method implemented in GROMACS, where the distance between structures was calculated as the RMSD over the heavy atoms of the residues in the OM binding site. Cutoff values were determined in each case to optimize the cluster partitioning and ranged from 0.10 to 0.13 nm.

## Results

### Modelling the CLD rotation in the recovery stroke

The motion of the CLD during the recovery stroke was modelled with SMD simulations. Two collective variables (CVs) were used to drive the transition: the distance between Q163 and L770 C_α_ atoms (Fig 1C), which describes the approach of the CLD to helix E (154–168) in the N-terminal domain, and the angle formed by A445, I462 and M776 C_α_ atoms (Fig 1D), which describes the rotational rearrangement of the CLD with respect to the rest of the protein.

The transition from the PR to the PPS state was induced by steering these CVs towards their target value in the PPS state (Table A in S1 Text). While the RMSD from either of the two states was not explicitly included as steering variable in the simulations, a steady decrease was observed in the RMSD of the CLD from the PPS state (pink in Fig 1E), with the structure increasingly deviating from the PR state at the same time (cyan in Fig 1E and 1F). At the end of the simulations, the relay helix, which is straight in the starting PR state, formed a kink around residue F494, which compares very well to the position of the kink in the reference PPS structure (pink in Fig 1G). A good overlap was also found between the SH1 helix structure at the end of the simulations (red) and its position in the reference structure (pink). Both the relay and the SH1 helix participate in OM binding and undergo significant changes during the recovery stroke [7]. Importantly, our simulations can reproduce well these changes without directly steering any of the atoms in these regions.

### Evolution of the OM binding pocket along the recovery stroke

The evolution of the binding pocket during the 200-ns PR-to-PPS SMD simulation was monitored by splitting the trajectory in batches and running the pocket analysis tool MDpocket [25] on each batch (Methods). While no kinetic information on pocket formation can be directly derived from the SMD simulations, the time evolution of the pocket volumes for the representative batches show that pockets are stably formed (volume > 0 nm^3^ for almost all the frames) within each batch (Fig F in S1 Text).

**Fig 2:**
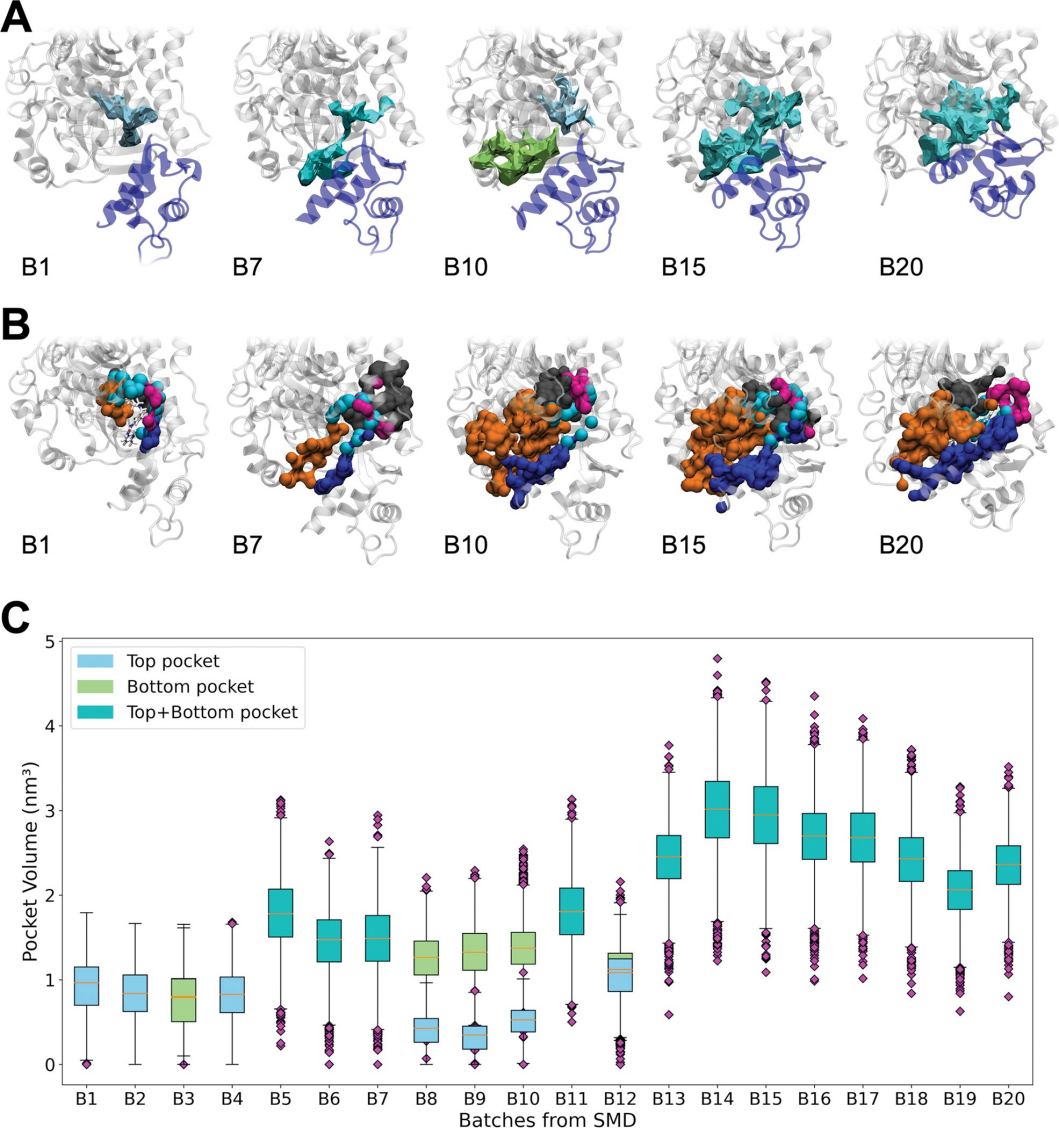
Change of shape and volume of the binding site along the recovery stroke. **A.** Isosurfaces connecting points with 30% pocket frequency as determined by MDpocket in 5 representative batches (B1, B7, B10, B15 and B20) from the 200-ns PR-to-PPS SMD trajectory. Three types of pockets were detected near the CLD (blue): a *top* pocket (light blue) in B1 and B10, a *bottom* pocket (green) in B10, and a *top+bottom* pocket where the two subpockets are joined together (cyan) in B7, B15 and B20. **B.** Surface representation of the binding region for representative structures of five batches along the 200-ns PR-to-PPS SMD simulation. The atoms lining the pockets are coloured according to their sub-domain: orange for the N-terminal domain, grey for L50K, magenta for the relay helix and blue for the converter and lever arm. Atoms from residues that are found to be part of a pocket in all the representative batches are highlighted as cyan spheres. The OM structure in the PR and PPS binding modes is superimposed to the B1 and B20 frames, respectively, and shown as sticks. **C.** Box plots of pocket volumes for twenty batches along the 200-ns PR-to-PPS SMD trajectory. For batches where the *top* and *bottom* pockets are both present but disjoint (B3, B8-10 and B12) two separate boxes are shown (one for each type of pocket). Each batch corresponds to approx. 5000 frames on average. The median (orange) and outliers (purple) are shown for each batch, with the length of the whiskers set to 1.5 IQR (inter-quartile range).

The pocket isofrequency surfaces (Fig 2A) show significant changes in the shape of the pocket during the transition. As the CLD rotates from the PR to the PPS state, the pocket extends to include new regions especially in the N-terminal domain (orange in Fig 2B) and the lever arm (blue). Correspondingly, the binding site volume becomes larger (Fig 2C). Moreover, while at the beginning of the transition (B1-B2) and in its second half (B13-B20) a continuous frequency isosurface is found, in the intermediate stages the surface is either split in two parts (as exemplified by B10 in Fig 2A) or features a narrow channel between the upper and lower regions (as shown for example by B7).

**Fig 3:**
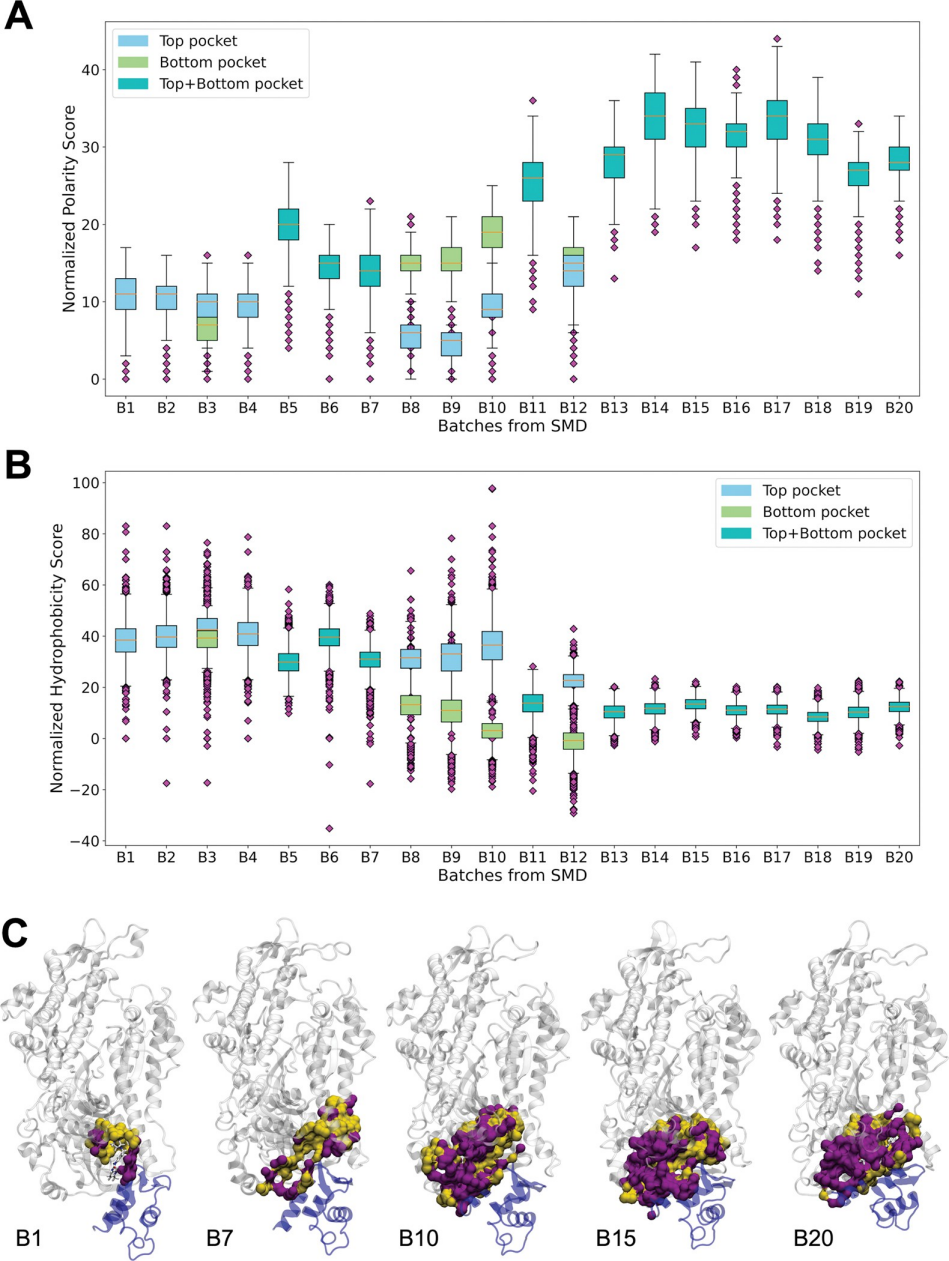
Change of binding site polarity along the recovery stroke. **A/B.** Box plots of the normalized polarity (A) and hydrophobicity (B) scores as determined by MDpocket for twenty batches of the 200-ns PR-to-PPS SMD trajectory. The polarity score increases during the simulation from ~11 to ~ 28, while the hydrophobicity one decreases from ~38 to ~12. **C.** Surface representation of the binding region for five representative batches coloured according to the residue type. The atoms lining the pockets are shown in yellow (hydrophobic residues) or purple (polar residues). The same structures and pockets represented in Fig 2B are used. The OM structure in the PR and PPS binding modes is superimposed to the B1 and B20 frames, respectively, and shown as sticks.

Analysis of the amino acidic composition of the binding site shows a significant increase in the polarity of the site (Fig 3A) and a corresponding decrease of its hydrophobic character (Fig 3B). Accordingly, the fraction of hydrophobic residues (yellow in Fig 3C) in the pocket decreases from 52% to 31%.

It is interesting to note that, despite the large changes in shape and composition of the binding site during the transition, a set of residues in the N-terminal domain (G119, L120 and F121), the relay helix (M493 and E497) and the converter (P710 and N711) is found to be part of the pocket in all the representative batches (cyan in Fig 2B and bold in Table D in S1 Text). Among these core residues, L120, E497 and N711 have been all found to be in contact with OM in both the PR [17] and PPS [14] states. On the other hand, SH1 residues V698, G701, I702 and C705 are part of the binding pocket only in the PR state (B1 batch, Table D in S1 Text), while regions in the N-terminal domain (~141–170), the L50K (~663–671) and the lever arm (~765–767 and 777–783) are involved in the pockets only in PPS and PPS-like states (B10, B15 and B20 batches, Table D in S1 Text).

The ability of the pockets to bind small molecules was investigated using FTMap [28], a tool that detects binding hotspots by identifying regions in the protein that can bind clusters of small molecule fragments (probes). Binding hotspots (consensus sites) were found by FTMap in the OM binding sites for all the representative batches (Fig G and Table E in S1 Text). The number of probe clusters, which has been found to be correlated with druggability, tends to increase for the top-ranking hotspots as the transition progresses towards the PPS state.

### Low energy intermediates along the transition

The free energy change along the transitions described by the PR-to-PPS and PPS-to-PR SMD simulations was estimated using US calculations (see Methods).

**Fig 4:**
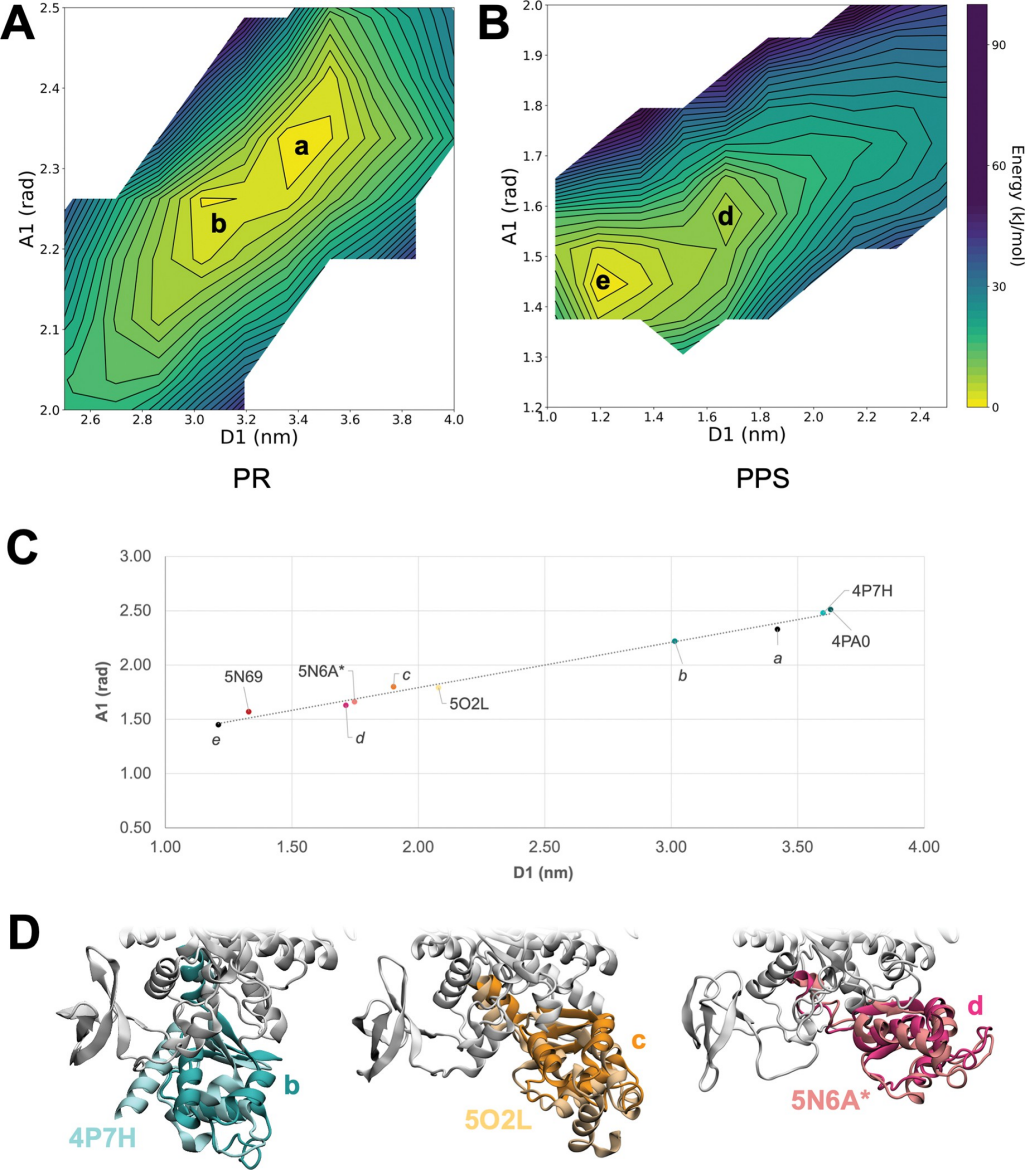
Intermediates along the recovery stroke. **A/B**. Free energy landscapes along the CVs with labelling of the minima *a*, *b*, *d* and *e*. Close-up views of the PR basin from the PR-to-PPS (A) and of the PPS basin from the PPS-to-PR (B) US calculations are shown. **C.** Scatterplot of the CV coordinates measured on X-ray structures and the *a*-*e* minima from US energy landscapes. 5N69 (chain B) [13]: bovine β-cardiac myosin (OM-bound), 5N6A (chain A) [13]: bovine β-cardiac myosin (OM-free), 5O2L (chain A) [29]: myosin VI (recovery stroke intermediate), 4P7H (chain A) [12]: human β-cardiac myosin (OM-free), 4PA0 (chain A) [12]: human β-cardiac myosin (OM-bound). For 5N6A, the structure with gaps completed and energy-minimised as described in reference [14] was used (5N6A*) since 5N6A does not contain one of the amino acids needed for the CV calculation (M776). For 5O2L, D1 was calculated as R136-F763 C_α_-atom distance, while A1 was calculated as the angle formed by Q441, I457 and I769 C_α_ atoms. **D.** Cartoon representation of intermediate structures *b* (left), *c* (middle) and *d* (right) superimposed to the X-ray structures (CLD only) closest to them. The CLD is highlighted in colour.

In the PR basin from the PR-to-PPS transition (Fig 4A), a global minimum (*a*) was found at D1 = 3.42 nm and A1 = 2.33 rad, which is consistent with the values sampled during unbiased simulations of the PR state (D1 = 3.32 nm and A1 = 2.30 rad at the end of the equilibration). A low-energy local minimum (*b*) was also found nearby (Fig 4A and Table F in S1 Text). The basin containing the two minima was quite extensive and shallow, and only a mild increase in energy (~ 17 kJ/mol) was observed up to halfway the transition to PPS (Fig H(A) in S1 Text).

The PPS basin from the PPS-to-PR transition displayed a global minimum at D1 = 1.21 nm and A1 = 1.45 rad (*e* in Fig 4B), which is consistent with the CV values observed in unbiased PPS MD simulations (D1 = 1.30 nm and A1 = 1.50 rad on average). A local minimum (*d*) was also found, but at a higher relative energy compared to what observed in the PR region (Table F in S1 Text).

At last, a high-energy intermediate (*c* in Fig H and Table F in S1 Text) was found in the PR-to-PPS landscape, far from either endpoint.

The difference between the full PR-to-PPS (Fig H(A) in S1 Text) and PPS-to-PR (Fig I(A) in S1 Text) landscapes indicates that the two sets of simulations are not sampling the same states. In particular, a minimum was not found for the target state of each transition, but only for the starting one. This suggests that a full conversion to the target state might require enhanced sampling along additional CVs and that the reliability of the present calculations is higher for regions close to the starting point of the transition compared to the target one.

As an independent assessment of the stability of the intermediate states *b*-*d*, representative structures were extracted from the US simulations sampling those regions (Methods) and unbiased MD simulations were run from them. Simulations starting from *b* structures sampled the region around that minimum and stayed close to the starting point (see Fig H(B) in S1 Text for a representative trajectory), providing further confirmation that *b* corresponds to a stable intermediate. For simulations starting from *c* and *d*, larger deviations from the initial structure were observed (Fig H(C-E) and I(B) in S1 Text) compared to *b*. For the *c* intermediate, this might be due to the high energy of this state coupled to modest energy barriers, so that in some of the trajectories the system could escape the minimum and start exploring the nearby lower energy regions (Fig H(C-D) in S1 Text).

The observed intermediate conformations *b*-*d* were also compared to X-ray myosin structures (Fig 4C and 4D). Plotting the CV values for X-ray and MD structures (Fig 4C) shows that all the considered experimental structures lie along a linear path connecting the two end points together with the MD-predicted ones, further validating our choice of CVs. Moreover, two experimental structures (5N6A [13] and the recently discovered recovery stroke intermediate 5O2L [29]) are found in vicinity of *d* and *c*. This supports the prediction that these two predicted states correspond to intermediates in the recovery stroke transition.

## Discussion

In this work, we investigate the changes in the structure and composition of a small-molecule binding site of human β-cardiac myosin during the recovery stroke transition. The myosin activator OM has been found to bind to this region in both endpoints of the transition (PR and PPS states), albeit with different binding affinity. We show that the PR and PPS binding pockets, rather than being distinct sites, can be seen as two different states of the same site, which interconvert into each other during the transition.

Previous studies modelling the recovery stroke in myosins have often used RMSD-based CVs [20,30,31] or transition pathways built using the coordinates of the whole protein [32,33]. Our CVs (a distance and an angle) involve only 5 C_α_ atoms and apart from the CV target values no other information was used to bias the simulations towards the target state. This choice was made to limit distortions of the binding site, which are possible when using RMSD-based CVs to drive a conformational change.

Our analysis shows significant changes in the shape, size and composition of the binding site along the recovery stroke, with the pocket becoming increasingly larger and more polar as the structure transitions towards the PPS conformation. These differences could be exploited to design compounds that can selectively target specific conformations. OM is able to bind both the PR and PPS state, with a partial selectivity towards the latter. This molecule binds close to the core residues (cyan in Fig 2B), which are consistently part of the binding site during the transition. Our data suggest that selectivity towards the PPS or PPS-like states could be improved by targeting regions farther from the core residues, which become part of the predicted pockets only in the later stages of the recovery stroke. On the other hand, increasing the hydrophobicity of the compounds and targeting the hydrophobic residues in the SH1 helix, which are not part of the binding site in the PPS state, could improve selectivity towards PR and PR-like states.

Our estimates of the free energy change along the transition indicate the presence of stable intermediates. These findings would need to be confirmed with additional sampling, possibly of regions far from the converter but allosterically coupled to it, in order to obtain a more accurate measurement of the free energy especially far from the starting point of the transition. However, two of the identified intermediates (*c* and *d*) are in good agreement with existing X-ray structures of myosin. These intermediates are close to two experimental structures in the CV space (Fig 4C), thus showing a similar degree of rotation of the CLD (Fig 4D). In particular, state *c* is close to a structure of myosin VI recently determined by X-ray crystallography and considered to be an intermediate along the recovery stroke of this protein[29]. While these two structures have a very similar angle A, some difference (~0.18 nm) was observed for the distance D. This might be due to inaccuracies in our free energy landscape or to intrinsic differences between the two proteins. It is interesting to note that unbiased trajectories starting from *c* are either stable or move towards 5O2L. The third predicted intermediate (*b*), while being the one with the highest confidence because of the stability of the unbiased MD simulations starting from it, does not have an experimental counterpart yet.

In conclusion, in this work we showed that a critical small-molecule binding site in cardiac myosin experiences considerable structural changes during the recovery stroke. We identified significant differences in the site shape, size and composition along the transition, which could be exploited to design compounds that can target different stages of the recovery stroke, thus expanding the therapeutic potential of myosin modulators. This is of particular interest for genetic muscle diseases (cardiac and skeletal) [34, 35] where myosin maladaptation varies from one patient to another and further personalised medicine is required.

## Supporting information

S1 Text Supplementary Information file. This file contains the following tables and figures: **Table A.** Overview of SMD simulations**; Table B.** Overview of US simulations–PR-to-PPS (recovery stroke)**; Table C.** Overview of US simulations–PPS-to-PR (inverse recovery stroke); **Table D.** Residue composition of the pockets detected by fpocket in the OM binding region along the recovery stroke**; Table E.** Binding hotspots found by FTMap in the OM binding site region for five representative batches; **Table F.** CV and energy values for the 5 minima found in the PR-to-PPS (*a-c*) and PPS-to-PR (*d* and *e*) energy landscapes; **Fig A.** Time evolution of selected distances during 300-ns PR and PPS MD trajectories; **Fig B.** Time evolution of selected angles during 300-ns PR and PPS MD trajectories; **Fig C.** Projection of PR and PPS 300-ns simulations on the 2D space for each of the 6 different CV combinations; **Fig D.** Time evolution of the CLD RMSD values from the reference PR and PPS structures during the 20-ns apoPR-to-PPS SMD trajectories for the six CV combinations; **Fig E.** Time evolution of the CLD RMSD values from the reference PR and PPS structures during the 200-ns apoPR-to-PPS SMD using D1, A1 and D2, A1; **Fig F.** Time evolution of the pocket volumes during the PR-to-PPS SMD trajectory; **Fig G.** Binding hotspots found by FTMap in the OM binding site region; **Fig H**. PR-to-PPS free energy landscape from US calculations; **Fig I**. PPS-to-PR free energy landscape from US calculations.
(DOCX)

## References

[1] Stank A, Kokh DB, Fuller JC, Wade RC. Protein Binding Pocket Dynamics. Acc Chem Res. 2016;49: 809–815. doi: 10.1021/acs.accounts.5b00516 27110726

[2] Beglov D, Hall DR, Wakefield AE, Luo L, Allen KN, Kozakov D, et al. Exploring the structural origins of cryptic sites on proteins. Proc Natl Acad Sci USA. 2018;115. doi: 10.1073/pnas.1711490115 29581267 PMC5899430

[3] Kuzmanic A, Bowman GR, Juarez-Jimenez J, Michel J, Gervasio FL. Investigating Cryptic Binding Sites by Molecular Dynamics Simulations. Acc Chem Res. 2020;53: 654–661. doi: 10.1021/acs.accounts.9b00613 32134250 PMC7263906

[4] Amaro RE, Baudry J, Chodera J, Demir Ö, McCammon JA, Miao Y, et al. Ensemble Docking in Drug Discovery. Biophysical Journal. 2018;114: 2271–2278. doi: 10.1016/j.bpj.2018.02.038 29606412 PMC6129458

[5] Pérot S, Sperandio O, Miteva MA, Camproux A-C, Villoutreix BO. Druggable pockets and binding site centric chemical space: a paradigm shift in drug discovery. Drug Discovery Today. 2010;15: 656–667. doi: 10.1016/j.drudis.2010.05.015 20685398

[6] Wassman CD, Baronio R, Demir Ö, Wallentine BD, Chen C-K, Hall LV, et al. Computational identification of a transiently open L1/S3 pocket for reactivation of mutant p53. Nat Commun. 2013;4: 1407. doi: 10.1038/ncomms2361 23360998 PMC3562459

[7] Robert-Paganin J, Pylypenko O, Kikuti C, Sweeney HL, Houdusse A. Force Generation by Myosin Motors: A Structural Perspective. Chem Rev. 2020;120: 5–35. doi: 10.1021/acs.chemrev.9b00264 31689091

[8] Preller M, Manstein DJ. Myosin Structure, Allostery, and Mechano-Chemistry. Structure. 2013;21: 1911–1922. doi: 10.1016/j.str.2013.09.015 24210227

[9] Malik FI, Hartman JJ, Elias KA, Morgan BP, Rodriguez H, Brejc K, et al. Cardiac Myosin Activation: A Potential Therapeutic Approach for Systolic Heart Failure. Science. 2011;331: 1439–1443. doi: 10.1126/science.1200113 21415352 PMC4090309

[10] Day SM, Tardiff JC, Ostap EM. Myosin modulators: emerging approaches for the treatment of cardiomyopathies and heart failure. Journal of Clinical Investigation. 2022;132: e148557. doi: 10.1172/JCI148557 35229734 PMC8884898

[11] Psotka MA, Gottlieb SS, Francis GS, Allen LA, Teerlink JR, Adams KF, et al. Cardiac Calcitropes, Myotropes, and Mitotropes. Journal of the American College of Cardiology. 2019;73: 2345–2353. doi: 10.1016/j.jacc.2019.02.051 31072579

[12] Winkelmann DA, Forgacs E, Miller MT, Stock AM. Structural basis for drug-induced allosteric changes to human β-cardiac myosin motor activity. Nat Commun. 2015;6: 7974. doi: 10.1038/ncomms8974 26246073 PMC4918383

[13] Planelles-Herrero VJ, Hartman JJ, Robert-Paganin J, Malik FI, Houdusse A. Mechanistic and structural basis for activation of cardiac myosin force production by omecamtiv mecarbil. Nat Commun. 2017;8: 190. doi: 10.1038/s41467-017-00176-5 28775348 PMC5543065

[14] Hashem S, Davies WG, Fornili A. Heart Failure Drug Modifies the Intrinsic Dynamics of the Pre-Power Stroke State of Cardiac Myosin. Journal of Chemical Information and Modeling. 2020;60: 6438−6446. doi: 10.1021/acs.jcim.0c00953 33283509

[15] Woody MS, Greenberg MJ, Barua B, Winkelmann DA, Goldman YE, Ostap EM. Positive cardiac inotrope omecamtiv mecarbil activates muscle despite suppressing the myosin working stroke. Nat Commun. 2018;9: 3838. doi: 10.1038/s41467-018-06193-2 30242219 PMC6155018

[16] Abraham MJ, Murtola T, Schulz R, Páll S, Smith JC, Hess B, et al. Gromacs: High performance molecular simulations through multi-level parallelism from laptops to supercomputers. SoftwareX. 2015;1–2: 19–25. doi: 10.1016/j.softx.2015.06.001

[17] Hashem S, Tiberti M, Fornili A. Allosteric modulation of cardiac myosin dynamics by omecamtiv mecarbil. PLoS computational biology. 2017;13: e1005826. doi: 10.1371/journal.pcbi.1005826 29108014 PMC5690683

[18] Lindorff-Larsen K, Piana S, Palmo K, Maragakis P, Klepeis JL, Dror RO, et al. Improved side-chain torsion potentials for the Amber ff99SB protein force field. Proteins: Structure, Function and Bioinformatics. 2010;78: 1950–1958. doi: 10.1002/prot.22711 20408171 PMC2970904

[19] Meagher KL, Redman LT, Carlson HA. Development of polyphosphate parameters for use with the AMBER force field. J Comput Chem. 2003;24: 1016–1025. doi: 10.1002/jcc.10262 12759902

[20] Woo H-J. Exploration of the conformational space of myosin recovery stroke via molecular dynamics. Biophysical Chemistry. 2007;125: 127–137. doi: 10.1016/j.bpc.2006.07.001 16889886

[21] Baldo AP, Tardiff JC, Schwartz SD. Mechanochemical Function of Myosin II: Investigation into the Recovery Stroke and ATP Hydrolysis. J Phys Chem B. 2020;124: 10014–10023. doi: 10.1021/acs.jpcb.0c05762 33136401 PMC7696650

[22] Baumketner A. The mechanism of the converter domain rotation in the recovery stroke of myosin motor protein: Converter Domain Rotation in Myosin II. Proteins. 2012;80: 2701–2710. doi: 10.1002/prot.24155 22855405 PMC3486948

[23] Ovchinnikov V, Trout BL, Karplus M. Mechanical Coupling in Myosin V: A Simulation Study. Journal of Molecular Biology. 2010;395: 815–833. doi: 10.1016/j.jmb.2009.10.029 19853615 PMC2813401

[24] Bussi G, Tribello GA. Analyzing and Biasing Simulations with PLUMED. Methods in Molecular Biology. 2019;2022: 529–578. doi: 10.1007/978-1-4939-9608-7_21 31396917

[25] Schmidtke P, Bidon-chanal A, Luque FJ, Barril X. MDpocket: Open-source cavity detection and characterization on molecular dynamics trajectories. Bioinformatics. 2011;27: 3276–3285. doi: 10.1093/bioinformatics/btr550 21967761

[26] Grossfield A. An implementation of WHAM: the Weighted Histogram Analysis Method Version 2.0.9.

[27] Daura X, Gademann K, Jaun B, Seebach D, van Gunsteren WF, Mark AE. Peptide Folding: When Simulation Meets Experiment. Angew Chem Int Ed. 1999;38: 236–240. doi: 10.1002/(SICI)1521-3773(19990115)38:1/2&lt;236::AID-ANIE236&gt;3.0.CO;2-M

[28] Kozakov D, Grove LE, Hall DR, Bohnuud T, Mottarella SE, Luo L, et al. The FTMap family of web servers for determining and characterizing ligand-binding hot spots of proteins. Nat Protoc. 2015;10: 733–755. doi: 10.1038/nprot.2015.043 25855957 PMC4762777

[29] Blanc F, Isabet T, Benisty H, Sweeney HL, Cecchini M, Houdusse A. An intermediate along the recovery stroke of myosin VI revealed by X-ray crystallography and molecular dynamics. Proc Natl Acad Sci USA. 2018;115: 6213–6218. doi: 10.1073/pnas.1711512115 29844196 PMC6004474

[30] Chakraborti A, Baldo AP, Tardiff JC, Schwartz SD. Investigation of the Recovery Stroke and ATP Hydrolysis and Changes Caused Due to the Cardiomyopathic Point Mutations in Human Cardiac β Myosin. J Phys Chem B. 2021;125: 6513–6521. doi: 10.1021/acs.jpcb.1c03144 34105970 PMC8281501

[31] Yu H, Ma L, Yang Y, Cui Q. Mechanochemical coupling in the myosin motor domain. I. Insights from equilibrium active-site simulations. PLoS computational biology. 2007;3: e21. 17291159 10.1371/journal.pcbi.0030021 PMC1796662

[32] Koppole S, Smith JC, Fischer S. The Structural Coupling between ATPase Activation and Recovery Stroke in the Myosin II Motor. Structure. 2007;15: 825–837. doi: 10.1016/j.str.2007.06.008 17637343

[33] Elber R, West A. Atomically detailed simulation of the recovery stroke in myosin by Milestoning. Proceedings of the National Academy of Sciences. 2010;107: 5001–5005. doi: 10.1073/pnas.0909636107 20194770 PMC2841901

[34] Jungbluth H, Treves S, Zorzato F, Sarkozy A, Ochala J, Sewry C, et al. Congenital myopathies: disorders of excitation–contraction coupling and muscle contraction. Nature Reviews Neurology. 2018;14: 151–167. doi: 10.1038/nrneurol.2017.191 29391587

[35] Lehman SJ, Crocini C, Leinwand LA. Targeting the sarcomere in inherited cardiomyopathies. Nat Rev Cardiol. 2022;19: 353–363. doi: 10.1038/s41569-022-00682-0 35304599 PMC9119933

