# Supplementary material for: Binding pocket dynamics along the recovery stroke of human β-cardiac myosin
Source: PLoS Comput Biol. 2023 May 18;19(5):e1011099. doi: 10.1371/journal.pcbi.1011099 (PMC10231807; doi:10.1371/journal.pcbi.1011099)
Supplement: S1 Text — This file contains the following tables and figures: Table A. Overview of SMD simulations; Table B. Overview of US simulations–PR-to-PPS (recovery stroke); Table C. Overview of US simulations–PPS-to-PR (inverse recovery stroke); Table D. Residue composition of the pockets detected by fpocket in the OM binding region along the recovery stroke; Table E. Binding hotspots found by FTMap in the OM binding site region for five representative batches; Table F. CV and energy values for the 5 minima found in the PR-to-PPS (a-c) and PPS-to-PR (d and e) energy landscapes; Fig A. Time evolution of selected distances during 300-ns PR and PPS MD trajectories; Fig B. Time evolution of selected angles during 300-ns PR and PPS MD trajectories; Fig C. Projection of PR and PPS 300-ns simulations on the 2D space for each of the 6 different CV combinations; Fig D. Time evolution of the CLD RMSD values from the reference PR and PPS structures during the 20-ns apoPR-to-PPS SMD trajectories for the six CV combinations; Fig E. Time evolution of the CLD RMSD values from the reference PR and PPS structures during the 200-ns apoPR-to-PPS SMD using D1, A1 and D2, A1; Fig F. Time evolution of the pocket volumes during the PR-to-PPS SMD trajectory; Fig G. Binding hotspots found by FTMap in the OM binding site region; Fig H. PR-to-PPS free energy landscape from US calculations; Fig I. PPS-to-PR free energy landscape from US calculations. (DOCX) [file pcbi.1011099.s001.docx]

Binding pocket dynamics along the recovery stroke of human β-cardiac myosin

Fariha Akter^1^, Julien Ochala^2,3^ and Arianna Fornili^1^*

1. Department of Chemistry, School of Physical and Chemical Sciences, Queen Mary University of London, London, E1 4NS, United Kingdom.
2. Department of Biomedical Sciences, University of Copenhagen, Blegdamsvej 3B, København N, DK-2200, Denmark
3. Centre of Human and Applied Physiological Sciences, King’s College London, London, SE1 9RT, United Kingdom.

*Corresponding author

E-mail: a.fornili@qmul.ac.uk

Table A. Overview of SMD simulations.

| *Starting state* | *Target state* | *CV1^a^* | *CV2^a^* | *Length* |
| --- | --- | --- | --- | --- |
| apoPR | apoPPS | D1 (3.7 ->1.3) | D2 (2.2 -> 3.2) | 20 ns |
| apoPR | apoPPS | D1 (3.7 ->1.3) | A1 (2.5 -> 1.5) | 20 ns |
| apoPR | apoPPS | D1 (3.7 ->1.3) | A2 (3.0 -> 2.3) | 20 ns |
| apoPR | apoPPS | D2 (2.2 -> 3.2) | A1 (2.5 -> 1.5) | 20 ns |
| apoPR | apoPPS | D2 (2.2 -> 3.2) | A2 (3.0 -> 2.3) | 20 ns |
| apoPR | apoPPS | A1 (2.5 -> 1.5) | A2 (3.0 -> 2.3) | 20 ns |
| apoPR | apoPPS | D1 (3.7 ->1.3) | A1 (2.5 -> 1.5) | 200 ns |
| apoPR | apoPPS | D2 (2.2 -> 3.2) | A1 (2.5 -> 1.5) | 200 ns |
| PR | PPS | D1 (3.3 -> 1.3) | A1 (2.3 -> 1.5) | 200 ns |
| PPS | PR | D1 (1.3 -> 3.3) | A1 (1.5 -> 2.3) | 200 ns |

^a^ CVs are indicated as D1 (Q163–L770 distance), D2 (I585–K707 distance), A1 (A445–I462–M776 angle) and A2 (L485–M493–E500 angle). Distances and angles are calculated using C_α_ atoms only. Values in parentheses indicate the starting and target values in nm (for distances) or radians (for angles).

| Table B. Overview of US simulations – PR-to-PPS (recovery stroke). | | | | | |
| --- | --- | --- | --- | --- | --- |
| *Window* | ***D1, A1 (nm, rad)^a^*** | ***Total length (ns)^b^*** | ***WHAM production (ns)^c^*** | |  |
| 1 | 3.74, 2.48 | 140 | 95 |  |  |
| 2 | 3.74, 2.23 | 100 | 92.9 |  |  |
| 3 | 3.13, 2.48 | 100 | 92 |  |  |
| 4 | 3.13, 2.23 | 110 | 90 |  |  |
| 5 | 3.13, 1.99 | 130 | 94 |  |  |
| 6 | 2.52, 2.23 | 150 | 90 |  |  |
| 7 | 2.52, 1.99 | 110 | 99 |  |  |
| 8 | 2.52, 1.74 | 120 | 91 |  |  |
| 9 | 1.91, 1.99 | 150 | 94 |  |  |
| 10 | 1.91, 1.74 | 160 | 97 |  |  |
| 11 | 1.91, 1.50 | 140 | 94 |  |  |
| 12 | 1.30, 1.74 | 110 | 94.5 |  |  |
| 13 | 1.30, 1.50 | 140 | 95 |  |  |
| 14 | 1.10, 1.30 | 140 | 92.5 |  |  |
| 15 | 2.83, 2.11 | 100 | 95 |  |  |
| 16 | 2.21, 1.87 | 130 | 97 |  |  |
| 17 | 1.61, 1.62 | 150 | 92 |  |  |
| 18 | 1.61, 1.50 | 100 | 100 |  |  |
| 19 | 1.20, 1.40 | 110 | 92.5 |  |  |
| 20 | 1.46, 1.62 | 100 | 100 |  |  |
| 21 | 2.21, 1.74 | 100 | 100 |  |  |
| 22 | 2.06, 1.87 | 100 | 100 |  |  |

^a^ Restraint values used for D1 and A1 in each US window.

^b^ Total length of the US simulation.

^c^ Number of ns at the end of the simulation used for the WHAM analysis. The initial part of the US simulation was discarded to allow for equilibration of the CVs.

**Table C.** Overview of US simulations – PPS-to-PR (inverse recovery stroke).

| *Window* | *D1, A1 (nm, rad)^a^* | *Total length (ns)^b^* | *WHAM production (ns)^c^* |
| --- | --- | --- | --- |
| 1 | 1.30, 1.50 | 130 | 99.5 |
| 2 | 1.30, 1.74 | 130 | 122.5 |
| 3 | 1.91, 1.50 | 100 | 93 |
| 4 | 1.91, 1.74 | 160 | 92 |
| 5 | 1.91, 1.99 | 120 | 94 |
| 6 | 2.52, 1.74 | 100 | 92 |
| 7 | 2.52, 1.99 | 130 | 107 |
| 8 | 2.52, 2.23 | 120 | 94.5 |
| 9 | 3.13, 1.99 | 100 | 96 |
| 10 | 3.13, 2.23 | 100 | 92 |
| 11 | 3.13, 2.48 | 105 | 93 |
| 12 | 3.74, 2.23 | 100 | 92.9 |
| 13 | 3.74, 2.48 | 120 | 99.1 |
| 14 | 1.61, 1.50 | 120 | 93 |
| 15 | 2.22, 1.87 | 120 | 95 |
| 16 | 2.22, 1.62 | 110 | 93 |
| 17 | 2.83, 1.99 | 110 | 95 |
| 18 | 2.83, 1.74 | 110 | 97 |
| 19 | 3.44, 2.36 | 120 | 92.5 |
| 20 | 3.44, 2.23 | 130 | 90 |
| 21 | 3.29, 2.05 | 100 | 100 |
| 22 | 3.00, 2.11 | 120 | 90 |
| 23 | 2.83, 2.11 | 130 | 92 |

^a^ Restraint values used for D1 and A1 in each US window.

^b^ Total length of the US simulation.

^c^ Number of ns at the end of the simulation used for the WHAM analysis. The initial part of the US simulation was discarded to allow for equilibration of the CVs.

**Table D.** Residue composition of the pockets detected by fpocket in the OM binding region along the recovery stroke.

| **B1^a^** | M90 | A91 | L96 | V101 | S118 | **G119** | **L120** | F121 | R147 | **M493** |
| --- | --- | --- | --- | --- | --- | --- | --- | --- | --- | --- |
|  | L496 | **E497** | E500 | V698 | G701 | I702 | C705 | **P710** | **N711** | R712 |
|  | K762 |  |  |  |  |  |  |  |  |  |
|  |  |  |  |  |  |  |  |  |  |  |
| **B7^a^** | M1 | E20 | R23 | L24 | K83 | F84 | I87 | A91 | M92 | L96 |
|  | **G119** | **L120** | S148 | N490 | **M493** | L496 | **E497** | Y501 | W508 | T509 |
|  | F510 | I511 | F513 | D516 | E700 | I704 | C705 | E707 | G708 | **P710** |
|  | **N711** | R712 | R721 | G768 | L770 | G771 | E774 | E775 |  |  |
|  |  |  |  |  |  |  |  |  |  |  |
| **B10^a^** | M1 | E5 | M6 | V8 | F9 | A13 | L16 | R17 | E20 | R23 |
|  | L24 | Q27 | K83 | F84 | I87 | E88 | D89 | M90 | A91 | M92 |
|  | L93 | L96 | Y117 | S118 | **G119** | **L120** | F121 | C122 | A141 | Y142 |
|  | K145 | R147 | S148 | E149 | A150 | P151 | S156 | I157 | N160 | Q163 |
|  | Y164 | F489 | N490 | H492 | **M493** | L496 | **E497** | F513 | H666 | P667 |
|  | H668 | F669 | R671 | N696 | G697 | V698 | G701 | I702 | C705 | **P710** |
|  | **N711** | R721 | G768 | L769 | L770 | G771 | L772 | E774 | E775 | R777 |
|  | D778 | E779 | R783 |  |  |  |  |  |  |  |
|  |  |  |  |  |  |  |  |  |  |  |
| **B15^a^** | M1 | E5 | R17 | K18 | E20 | E22 | R23 | L24 | A26 | Q27 |
|  | K83 | D85 | K86 | I87 | E88 | D89 | M90 | A91 | M92 | L96 |
|  | V101 | R108 | Y117 | S118 | **G119** | **L120** | F121 | C122 | Y142 | K145 |
|  | K146 | R147 | S148 | A150 | S156 | I157 | N160 | Q163 | Y164 | F489 |
|  | H492 | **M493** | **E497** | Y501 | F510 | H666 | H668 | F669 | V698 | G701 |
|  | I702 | I704 | C705 | G708 | F709 | **P710** | **N711** | R712 | I713 | D717 |
|  | Q720 | R721 | Y722 | G768 | L770 | G771 | E774 | E775 | R777 | D778 |
|  | E779 | R783 |  |  |  |  |  |  |  |  |
|  |  |  |  |  |  |  |  |  |  |  |
| **B20^a^** | M1 | E5 | Y117 | **G119** | **L120** | F121 | C122 | Y142 | R143 | G144 |
|  | K145 | K146 | R147 | S148 | GLU149 | A150 | S156 | I157 | D159 | N160 |
|  | Q163 | Y164 | T167 | D168 | E170 | H492 | **M493** | L496 | **E497** | GLU500 |
|  | R663 | T665 | H666 | P667 | H668 | F669 | V670 | **P710** | **N711** | R712 |
|  | I713 | L714 | D717 | R721 | LYS762 | F765 | K766 | A767 | G768 | L769 |
|  | L770 | G771 | L772 | E774 | E775 | R777 | D778 | E779 | R783 |  |

^a^Representative batches extracted from the 200-ns PR-to-PPS SMD simulation. Residues that are found to be part of the pockets in all the analysed batches are highlighted in bold.

**Table E.** Binding hotspots found by FTMap in the OM binding site region for five representative batches.

|  | **Ranking^a^** | **Number of probe clusters^b^** |
| --- | --- | --- |
| **B1** | 3 | 10 |
|  | 5 | 6 |
|  | 12 | 2 |
|  | 14 | 1 |
| **B7** | 5 | 6 |
| **B10** | 1 | 12 |
|  | 2 | 10 |
|  | 4 | 7 |
|  | 10 | 3 |
|  | 14 | 2 |
| **B15** | 0 | 16 |
|  | 1 | 16 |
|  | 3 | 8 |
|  | 4 | 6 |
|  | 8 | 4 |
|  | 12 | 2 |
| **B20** | 1 | 19 |
|  | 4 | 6 |
|  | 7 | 4 |
|  | 8 | 3 |
|  | 10 | 3 |
|  | 11 | 2 |

^a^FTMap ranking (starting from 0) of hotspots (consensus sites) found in the representative structure of batches B1, B7, B10, B15 and B20. The same colour scheme is used as in Fig. G.

^b^Number of probe clusters for each consensus site. The number of clusters is expected to be proportional to the druggability of a site.

**Table F.** CV and energy values for the 5 minima found in the PR-to-PPS (*a-c*) and PPS-to-PR (*d* and *e*) energy landscapes.

| *Minimum* | *D1 (nm)* | *A1 (rad)* | *Energy (kJ/mol)* |
| --- | --- | --- | --- |
| *a* | 3.42 | 2.33 | 0.00 |
| *b*^1^ | 3.01 | 2.22 | 1.80 |
| *c*^1^ | 1.90 | 1.80 | 35.54 |
| *d*^2^ | 1.71 | 1.63 | 6.52 |
| *e* | 1.21 | 1.45 | 0.00 |

^1^The energy value is relative to *a.*

^2^The energy value is relative to *e.*

**
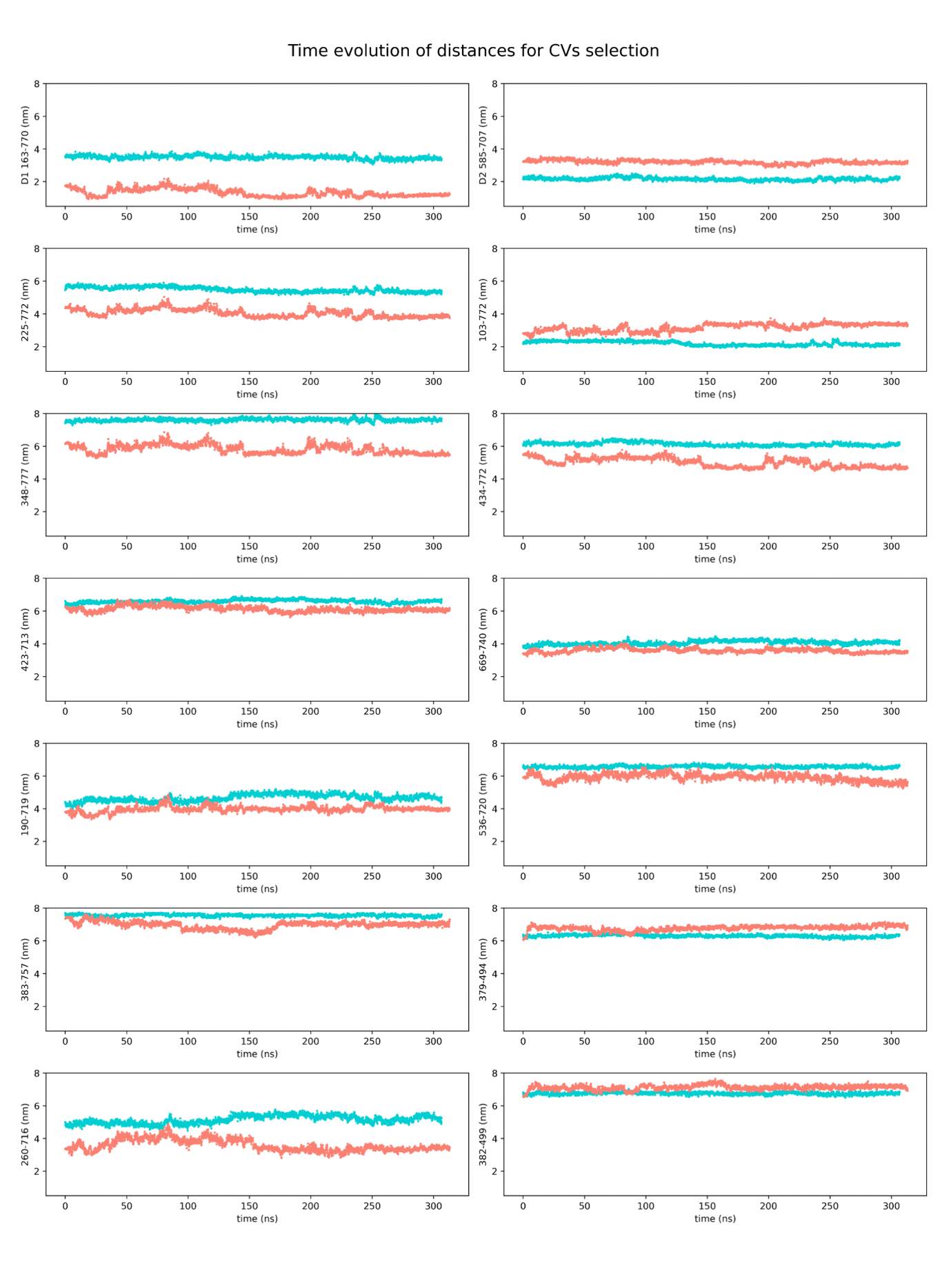
**

**Fig A.** Time evolution of selected distances (nm) during the 300-ns PR (cyan) and PPS (pink) MD trajectories from Ref. 1 and 2.

**
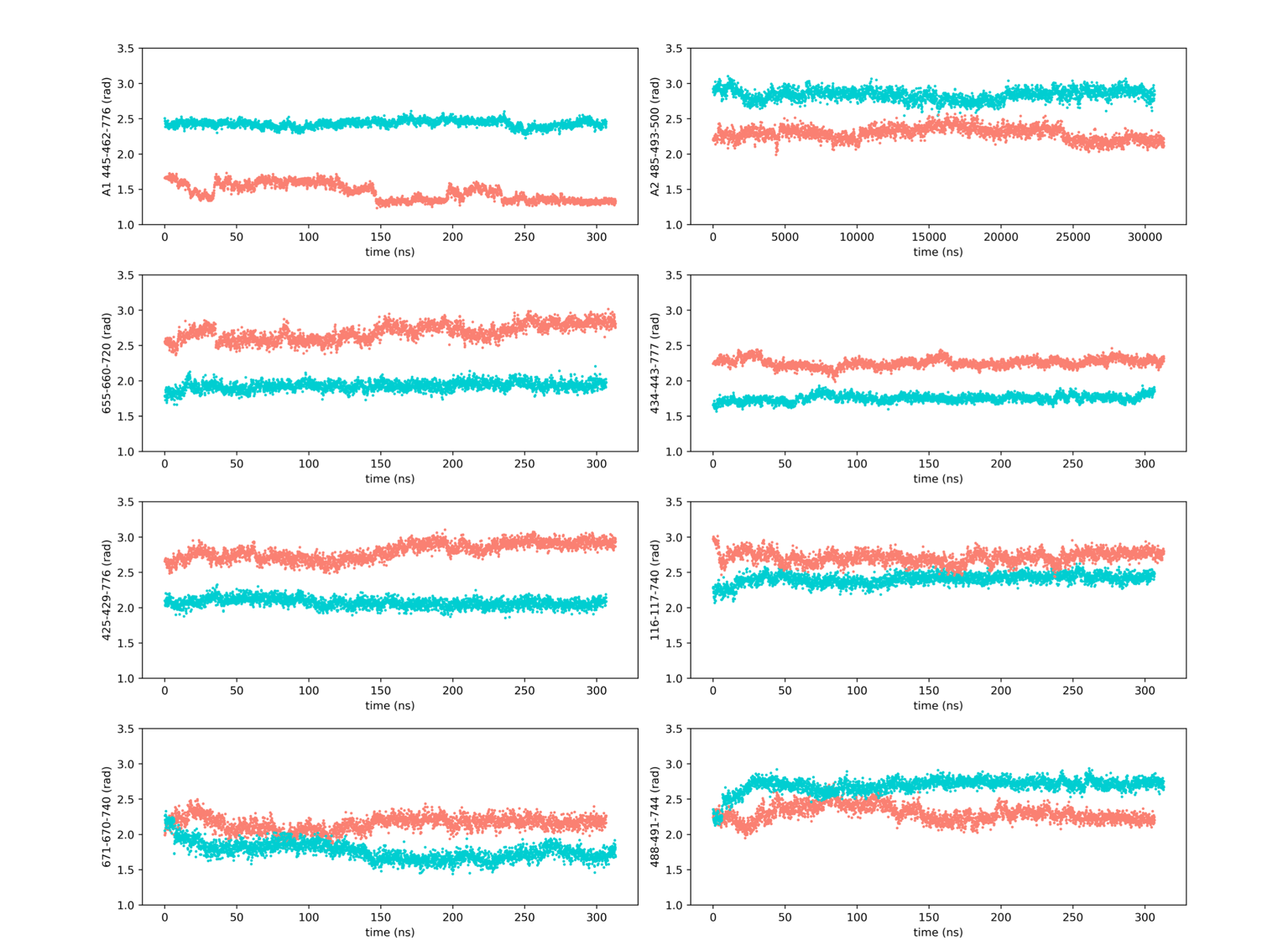
**

**Fig B.** Time evolution of selected angles during the 300-ns PR (cyan) and PPS (pink) MD trajectories from Ref. 1 and 2.


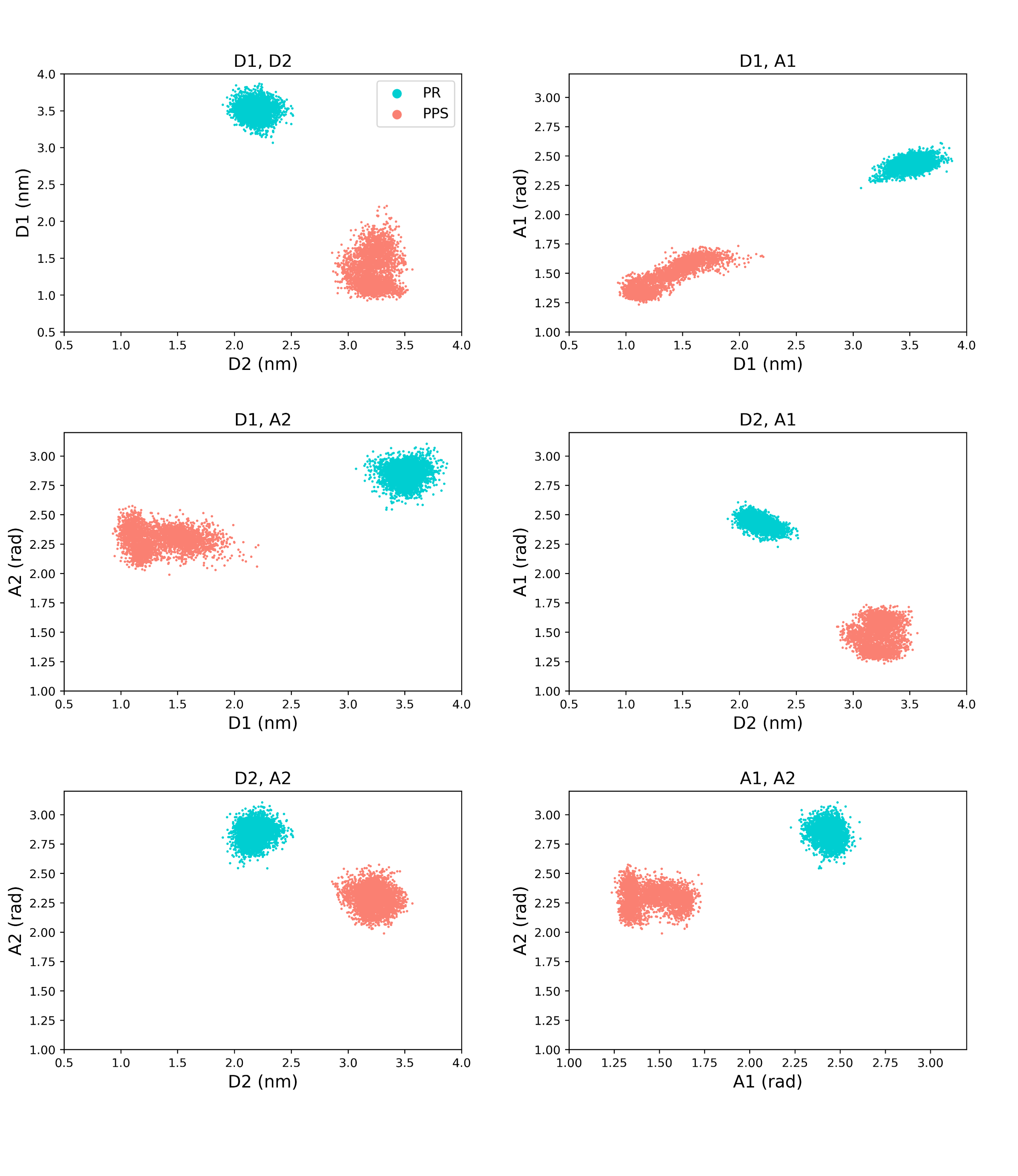


**Fig C.** Projection of the PR (cyan) and PPS (pink) 300-ns simulations (from Ref. 1 and 2 in the main text) on the 2D space for each of the 6 different CV combinations.


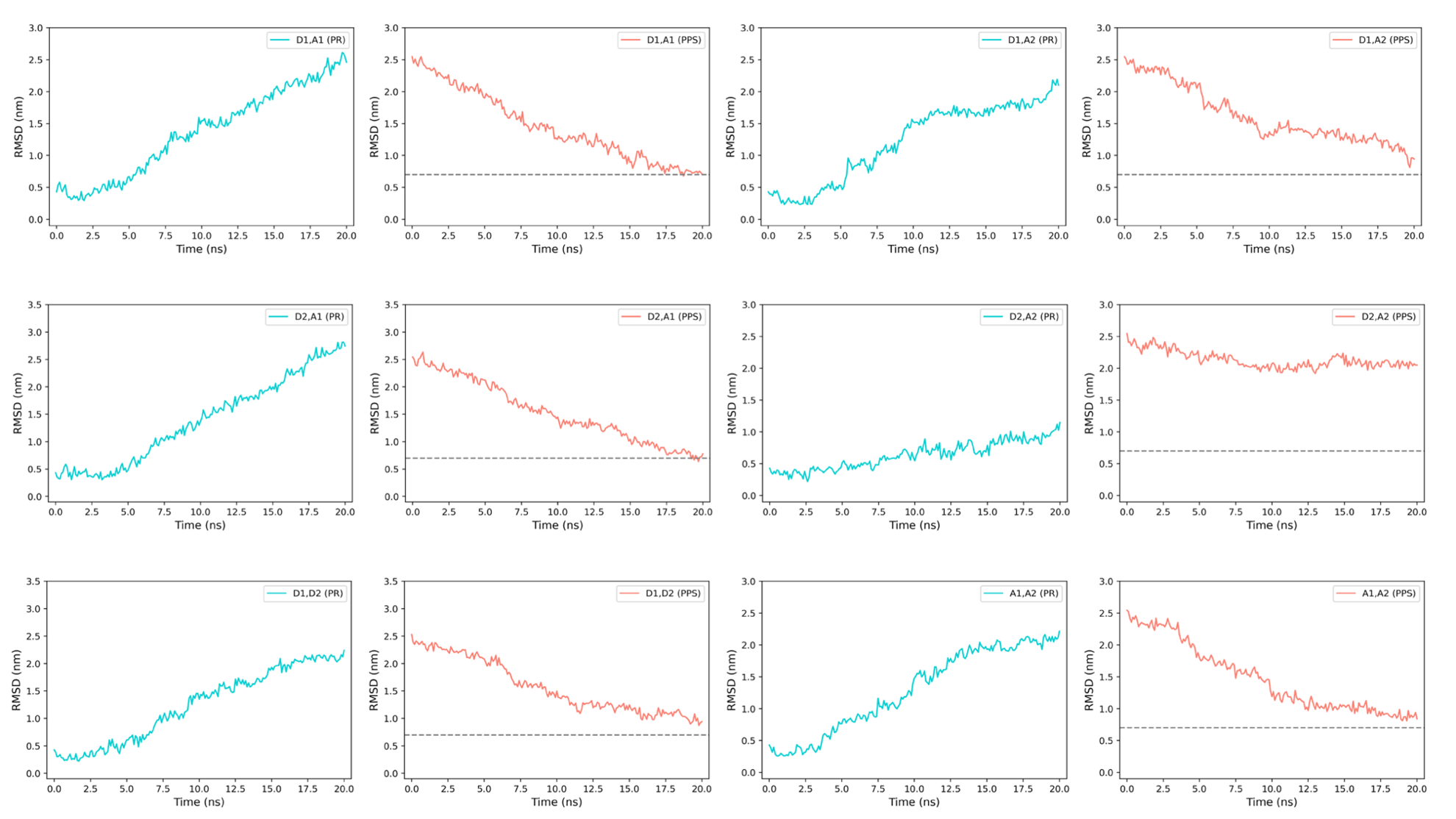


**Fig D.** Time evolution of the CLD RMSD values from the reference PR (cyan) and PPS (pink) structures during the 20-ns apoPR-to-PPS SMD trajectories for the six CV combinations. The RMSD was calculated over the C_α_ atoms of the CLD domain after a best-fit superimposition of the C_α_ atoms of the rest of the protein. A grey dotted line is added at 0.7 nm as an eye guide.


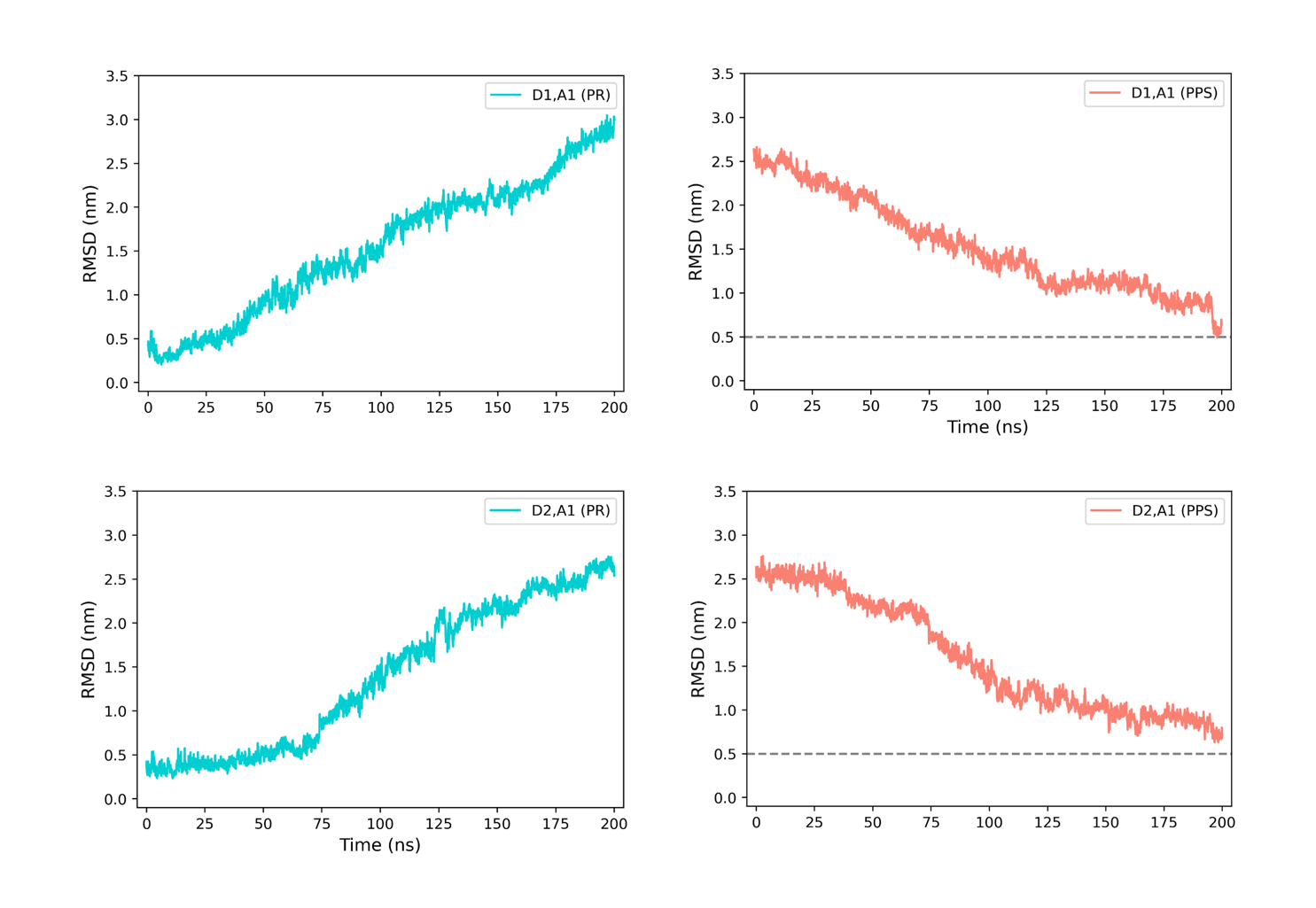


**Fig E.** Time evolution of the CLD RMSD values from the reference PR (cyan) and PPS (pink) structures during the 200-ns apoPR-to-PPS SMD using D1, A1 and D2, A1. The RMSD was calculated over the C_α_ atoms of the CLD domain after a best-fit superimposition of the C_α_ atoms of the rest of the protein. A grey dotted line is added at 0.5 nm as an eye guide.


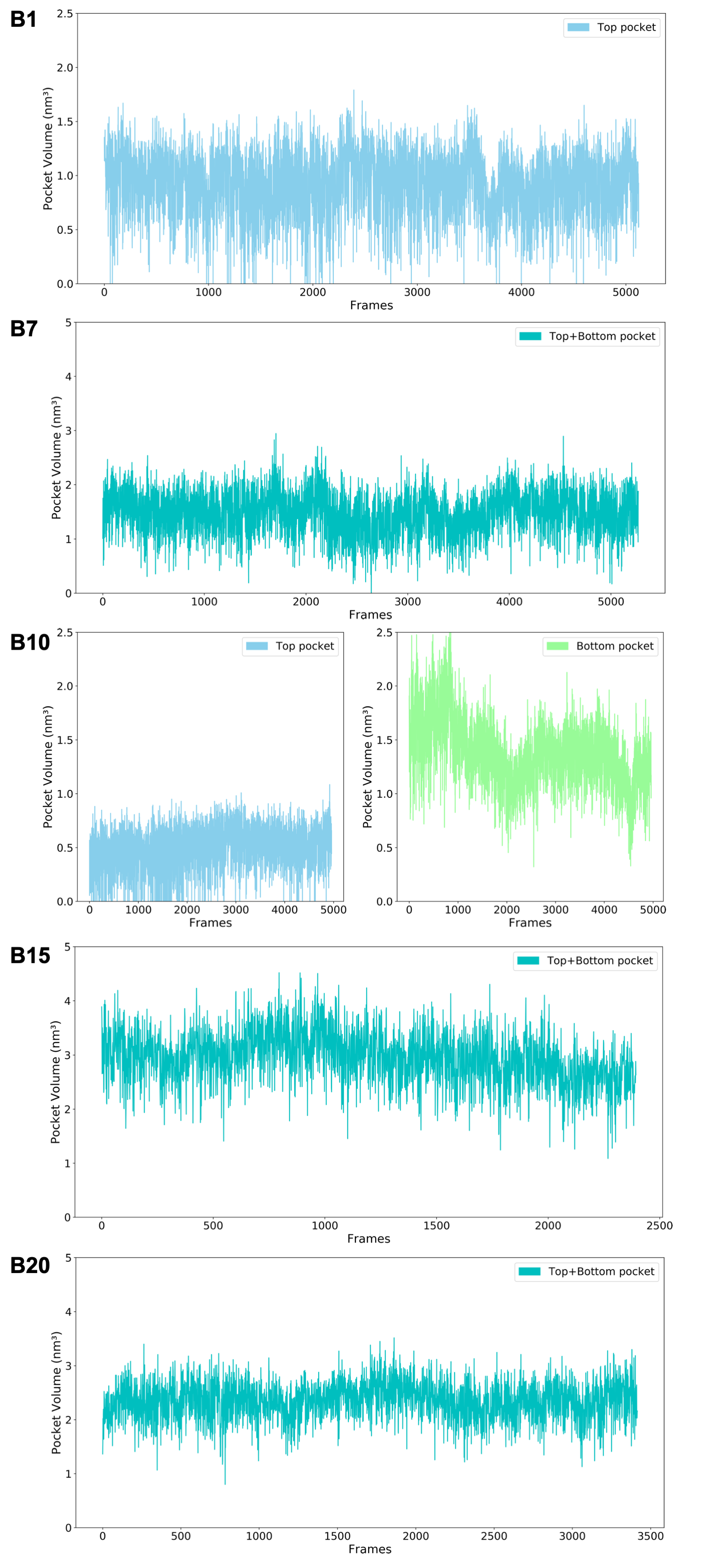


**Fig F.** Time evolution of the pocket volumes during the PR-to-PPS SMD trajectory. Volumes of *top* (light blue), *bottom* (green) and *top+bottom* (cyan) pockets are reported for representative batches along the simulation.


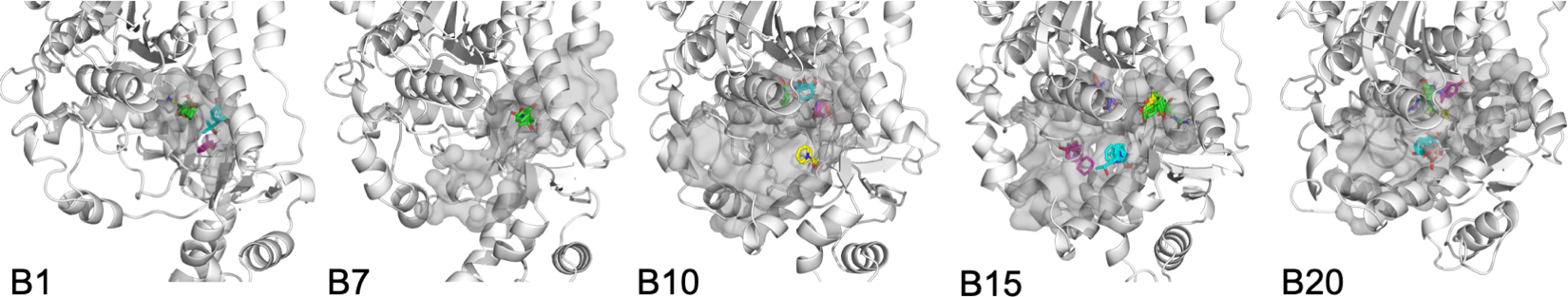


**Fig G.** Binding hotspots found by FTMap in the OM binding site region. FTMap was run using default parameters on the representative structures of batches B1, B7, B10, B15 and B20. Binding hotspots are indicated by showing the probe cluster representatives (coloured sticks) of the consensus sites identified by FTMap in the pockets detected by fpocket (grey surface). The colour scheme is the same as that used for Table E.


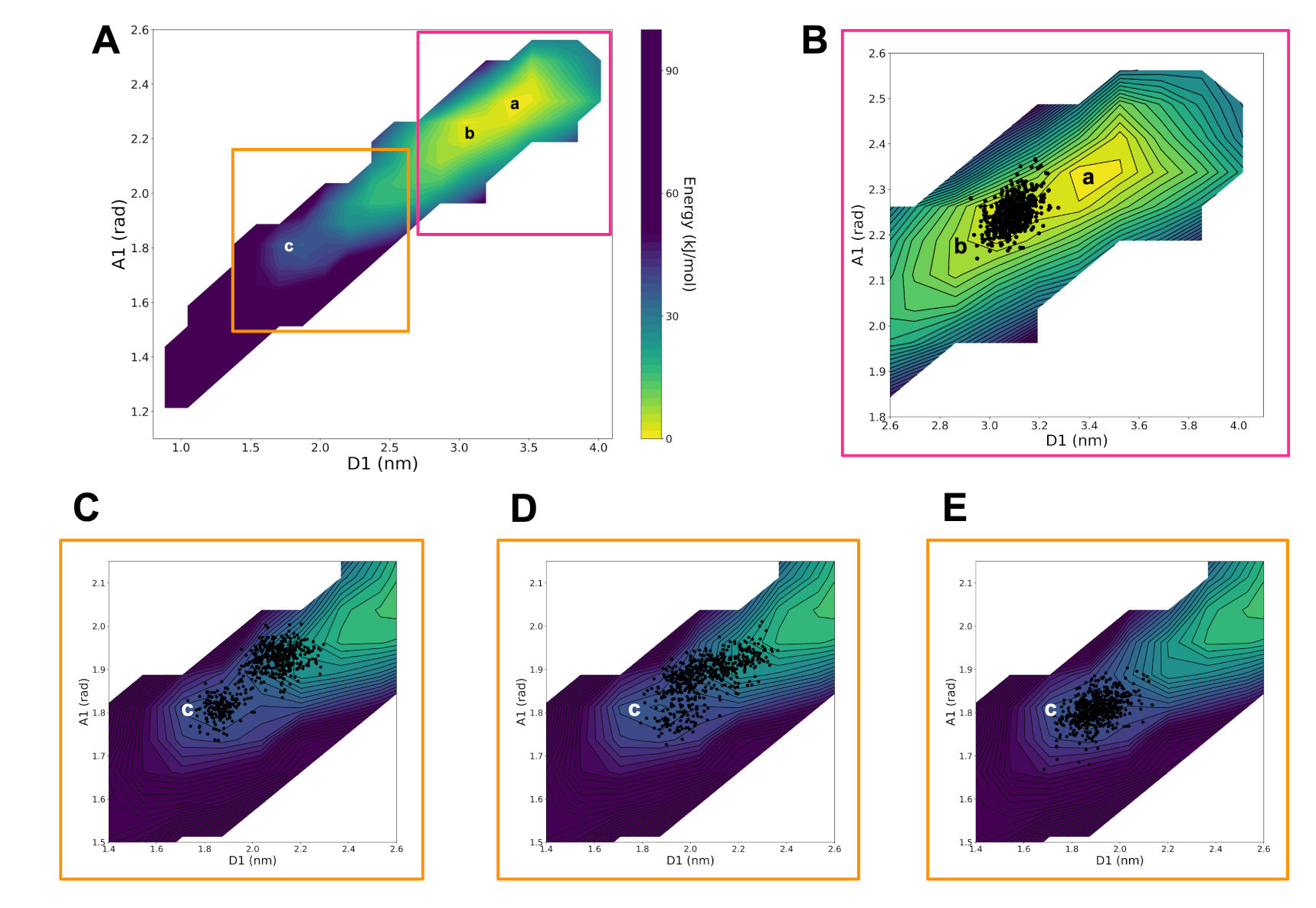


**Fig H. A.** PR-to-PPS free energy landscape from US calculations. **B-E.** Close up view of the PR basin (B) and of the region around the intermediate *c* (C-E). MD trajectories (50-ns) starting from *b* (representative structure of the most populated cluster in *b*) and *c* (representative structures of the 1^st^ (C), 2^nd^ (D) and 3^rd^ (E) most populated clusters in *c*) are projected on the landscape (black dots).

**
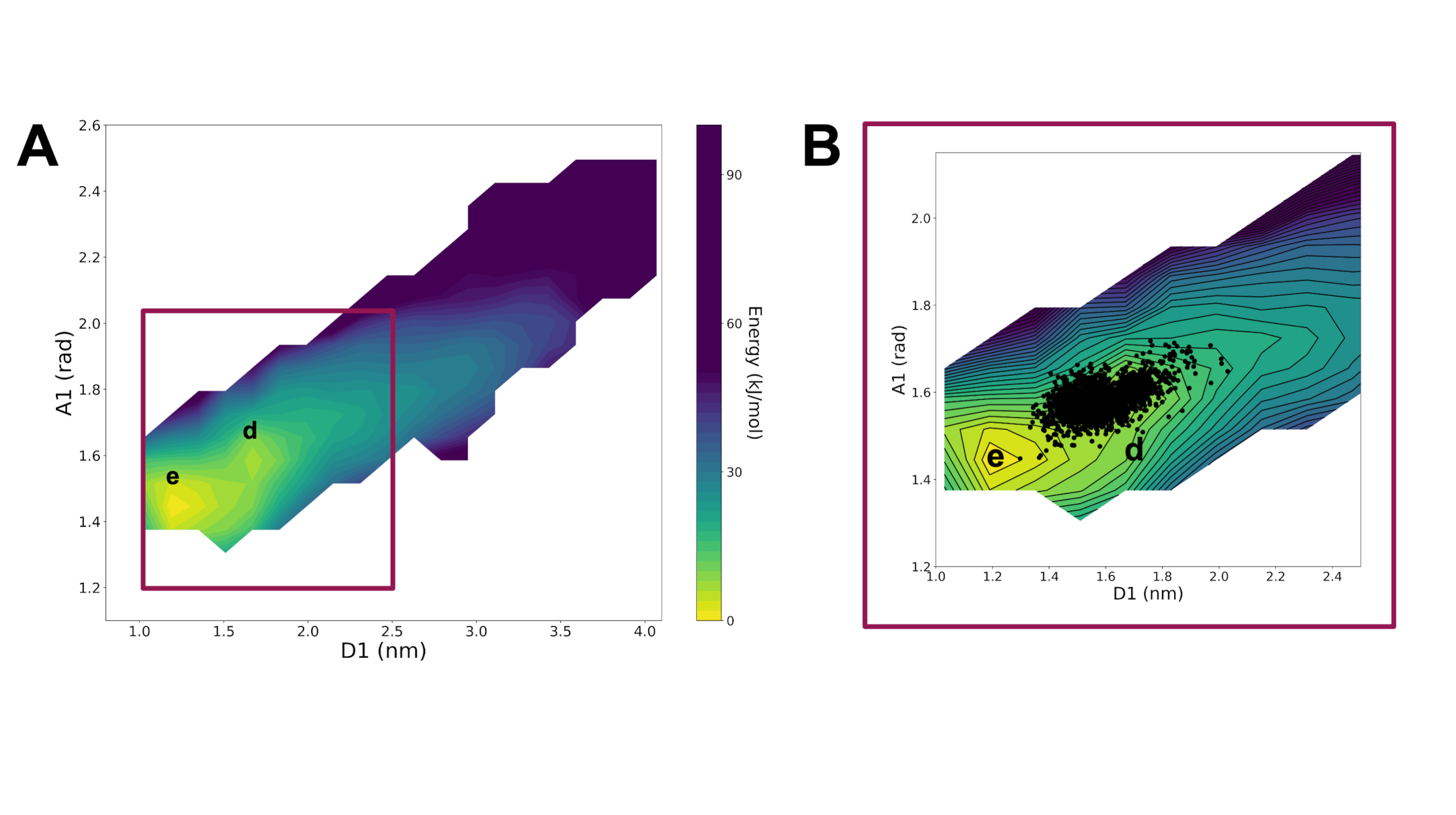
**

**Fig I. A.** PPS-to-PR free energy landscape from US calculations. **B.** Close up view of the PPS basin. A 150-ns MD trajectory starting from *d* (representative of the most populated cluster in *d*) is projected on the landscape (black dots).

**References**

1. Hashem S, Davies WG, Fornili A. Heart Failure Drug Modifies the Intrinsic Dynamics of the Pre-Power Stroke State of Cardiac Myosin. Journal of Chemical Information and Modeling. 2020;60: 6438−6446.

2. Hashem S, Tiberti M, Fornili A. Allosteric modulation of cardiac myosin dynamics by omecamtiv mecarbil. PLoS computational biology. 2017;13: e1005826.
